# Usability, Acceptability, and Barriers to Implementation of a Collaborative Agenda-Setting Intervention (CASI) to Promote Person-Centered Ovarian Cancer Care: Development Study

**DOI:** 10.2196/66801

**Published:** 2025-03-10

**Authors:** Rachel A Pozzar, James A Tulsky, Donna L Berry, Jeidy Batista, Paige Barwick, Charlotta J Lindvall, Patricia C Dykes, Michael Manni, Ursula A Matulonis, Nadine J McCleary, Alexi A Wright

**Affiliations:** 1 Department of Medical Oncology, Dana-Farber Cancer Institute, 450 Brookline Avenue, BP1143, Boston, MA, 02215, United States, 1 8572150743, 1 6175828550; 2 Department of Medicine, Harvard Medical School, Boston, MA, United States; 3 Department of Medicine, Brigham and Women’s Hospital, Boston, MA, United States; 4 Department of Supportive Oncology, Dana-Farber Cancer Institute, Boston, MA, United States; 5 Department of Biobehavioral Nursing and Health Informatics, University of Washington, Seattle, WA, United States; 6 Department of Informatics and Analytics, Dana-Farber Cancer Institute, Boston, MA, United States

**Keywords:** ovarian neoplasm, ovarian cancer, cancer, oncology, oncologist, metastases, communication, physician-patient relations, electronic health record, EHR, electronic medical record, EMR, implementation science, digital, digital health, digital technology, digital intervention, mobile phone

## Abstract

**Background:**

People with advanced ovarian cancer and their caregivers report unmet supportive care needs. We developed a Collaborative Agenda-Setting Intervention (CASI) to elicit patients’ and caregivers’ needs through the patient portal before a clinic visit and to communicate these needs to clinicians using the electronic health record.

**Objective:**

We aimed to assess the usability and acceptability of the CASI and identify barriers to and facilitators of its implementation.

**Methods:**

We recruited English- and Spanish-speaking patients, caregivers, and clinicians from the gynecologic oncology program at a comprehensive cancer center. Participants used the CASI prototype and then completed individual cognitive interviews and surveys. We assessed usability with the System Usability Scale (scores range 0‐100, scores ≥70 indicate acceptable usability) and acceptability with the Acceptability of Intervention Measure and Intervention Appropriateness Measure (scores for both measures range from 1 to 5, higher scores indicate greater acceptability). Interviews were audio recorded, transcribed, and analyzed using directed content analysis. Domains and constructs from the Consolidated Framework for Implementation Research comprised the initial codebook. We analyzed survey data using descriptive statistics and compared usability and acceptability scores across patients, caregivers, and clinicians using analyses of variance.

**Results:**

We enrolled 15 participants (5 patients, 5 caregivers, and 5 clinicians). The mean System Usability Scale score was 72 (SD 16). The mean Acceptability of Intervention Measure and Intervention Appropriateness Measure scores were 3.9 (SD 1.0) and 4.1 (SD 0.8), respectively. Participants viewed the CASI content and format positively overall. Several participants appreciated the CASI’s integration into the clinical workflow and its potential to increase attention to psychosocial concerns. Suggestions to refine the CASI included removing redundant items, simplifying item language, and adding options to request a conversation or opt out of supportive care referrals. Key barriers to implementing the CASI include its complexity and limited resources available to address patients’ and caregivers’ needs.

**Conclusions:**

The CASI is usable and acceptable to patients with advanced ovarian cancer, caregivers, and clinicians. We identified several barriers to and facilitators of implementing the CASI. In future research, we will apply these insights to a pilot randomized controlled trial to assess the feasibility of comparing the CASI to usual care in a parallel group-randomized efficacy trial.

## Introduction

Patients undergoing treatment for advanced ovarian cancer commonly experience burdensome disease- and treatment-related symptoms [1] followed by cancer recurrence after treatment completion [2]. Challenged to adapt to a chronic, life-limiting condition, more than two-thirds of patients with advanced ovarian cancer and their caregivers report at least 1 moderate-to-high unmet supportive care need [3]. Such needs negatively impact patients’ and caregivers’ health-related quality of life and are associated with a higher likelihood that patients will require emergency department visits and hospitalizations [4]. In 2022, a multidisciplinary expert panel recommended the provision of individualized and timely resources to address the unmet supportive care needs of patients with advanced ovarian cancer and their caregivers [5]. Likewise, health authorities in the United States, Canada, and the United Kingdom have advocated for the delivery of person-centered (alternatively, “patient-centered”) cancer care [6-8].

Person-centered care entails eliciting and responding to patients’ and caregivers’ goals, values, and preferences in a system that supports high quality communication between patients, caregivers, and clinicians [9,10]. Research suggests the provision of person-centered care has the potential to improve health outcomes in patients with cancer [11]. In our prior work, we found that communication that fosters healing patient-clinician relationships is associated with better social and family well-being and lower symptom burdens among people with ovarian cancer [12,13]. Patients in these studies wanted their clinicians to be proactive and attentive to patients’ psychosocial concerns and other supportive care needs [13]. However, time constraints, medical complexity, and inadequate resources for follow-up care may challenge clinicians to identify and manage nonmedical needs routinely [14-16].

To overcome these barriers, we used a design thinking approach to develop a Collaborative Agenda-Setting Intervention (CASI) to promote person-centered ovarian cancer care [17]. The CASI is a patient portal- and electronic health record (EHR)–integrated tool that aims to improve patient and caregiver well-being by routinely eliciting patients’ and caregivers’ values, preferences, and supportive care needs. The CASI supports agenda-setting and person-centered communication between patients, caregivers, and clinicians. The American Society of Clinical Oncology [18] and the European Society for Medical Oncology [19] have published guidelines for person-centered communication which direct clinicians to routinely set an agenda for visits by sharing their goals and eliciting topics that patients and caregivers wish to address. In the primary care setting, agenda-setting has been shown to increase person-centered care without prolonging visit duration [20] and to reduce clinician burden by preventing late-breaking concerns [21]. To date, however, research on agenda-setting interventions in cancer care is limited. The purpose of this study, therefore, was to assess the usability and acceptability of the CASI and identify barriers to and facilitators of implementing the CASI in this setting.

## Methods

### Study Design

We conducted a cross-sectional study using qualitative and quantitative methods. Our approach to data collection and analysis was guided by principles of design thinking [17] and the Consolidated Framework for Implementation Research (CFIR) [22]. According to the CFIR, the likelihood of successful implementation is affected by 5 domains: the inner setting (ie, the setting in which the innovation is being implemented), the outer setting (ie, the community or system in which the inner setting exists), the characteristics of the innovation itself, the characteristics of the individuals who will interact with the innovation, and the process by which the innovation is implemented [22]. We tested the CASI prototype and elicited stakeholder perspectives on each of the CFIR domains.

### Recruitment

We recruited participants from the gynecologic oncology clinic of a National Cancer Institute–designated comprehensive cancer center. Patients were eligible if they were English- or Spanish-speaking adults with stage III, stage IV, or recurrent ovarian cancer. Caregivers were eligible if they were English- or Spanish-speaking adults who self-identified as the caregiver of a person with stage III, stage IV, or recurrent ovarian cancer. Enrollment in the cancer center’s patient portal was not required for study participation. Patients and caregivers were purposively sampled to ensure representation of diverse demographic groups. We approached potentially eligible patients and caregivers in person during regularly scheduled clinic visits. Clinicians were eligible if they were an oncologist or advanced practice provider who cared for at least 4 outpatients per month with stage III, stage IV, or recurrent ovarian cancer. We introduced this study at a weekly provider meeting, then approached individual clinicians via email.

### Ethical Considerations

All participants provided written informed consent and received US $25 upon data collection. Study data were deidentified before analysis. The Dana-Farber Cancer Institute Institutional Review Board approved all study procedures (protocol #21‐322).

### Intervention Characteristics

**Figure 1.**
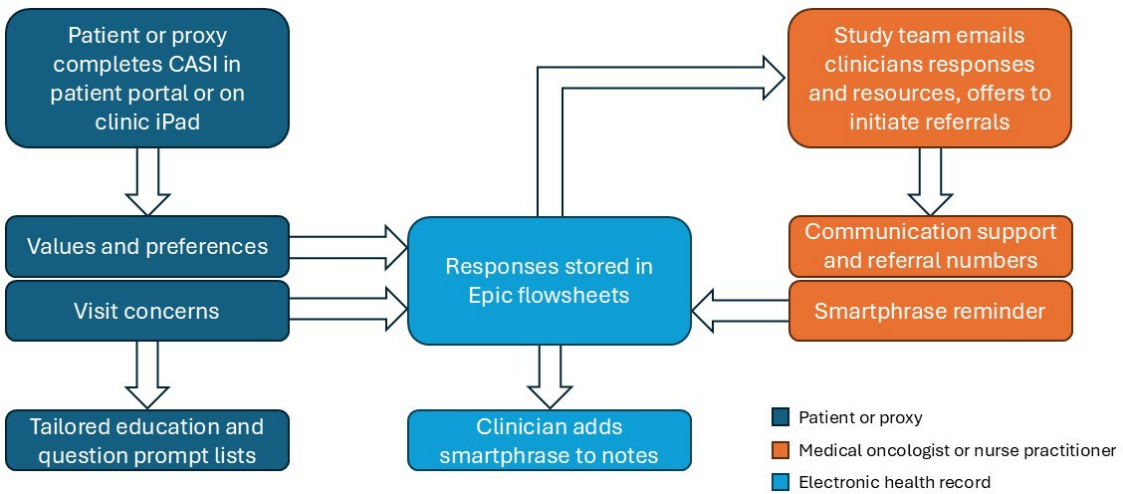
CASI schema. CASI: Collaborative Agenda-Setting Intervention.

An intervention schema is provided in (Figure 1). The CASI has been integrated into Epic (Epic Systems Corporation), which is the most widely used EHR system in the United States [23]. The CASI is currently available in English and Spanish and has an English-language Flesch-Kincaid reading level of grade 6.9. Patients complete the CASI through the patient portal, which can be accessed on any smartphone, tablet, laptop, or desktop computer. Patients without a personal device may complete the CASI on a tablet provided by the clinic at the time of check-in. Caregivers with proxy access to the patient portal may complete the CASI in the same fashion as patients. Clinicians access the patient’s or caregiver’s responses to the CASI in Epic hyperspace, which is the clinician-facing EHR platform.

Patients and caregivers are prompted through the patient portal to complete the CASI no more than 7 days before an upcoming clinic visit. Completing the CASI involves responding to 2 questionnaires as frequently as once every 3 weeks. The first questionnaire includes items about values, preferences, and communication needs. Selected items were derived from the Control Preferences Scale [24], which is a validated measure of patients’ preferred level of involvement in medical decision-making, and the SHARE questionnaire [25], a communication intervention designed to elicit patients’ preferences and goals. Patients and caregivers complete the first questionnaire the first time they use the CASI. Thereafter, patients and caregivers have the option to update their responses but are not required to repeat the questionnaire. The second questionnaire includes items derived from the National Comprehensive Cancer Network Distress Thermometer Problem List [26], which asks respondents to select the physical, emotional, social, practical, spiritual, and other concerns experienced during the past 7 days. Patients and caregivers complete the second questionnaire every time they use the CASI. Patients and caregivers receive standardized question prompt lists through the patient portal for each of the concerns they identify. In addition, a member of this study’s team assists with follow-up navigation and initiating referrals when warranted.

Responses to both questionnaires are stored in Epic and associated with the appropriate upcoming clinical encounter. Clinicians can view CASI questionnaire responses in the rooming tab of the visit note (which contains patient-reported symptoms, vital signs, and information collected by the clinic assistant before the patient is seen by the physician or advanced practice provider), during chart review, or by using a smart phrase to populate the narrative history of present illness with the most recent CASI questionnaire responses. When a patient or caregiver completes the CASI, a member of this study’s team emails the clinician a summary of the CASI responses, offers to initiate referrals, shares links to evidence-based communication guidance, and reminds the clinician how to use the CASI smart phrase to populate their visit note.

### Data Collection

#### Participant Characteristics

All participants self-reported age, gender, ethnicity, and race. Patients and caregivers self-reported marital status, annual household income, and educational attainment. Caregivers self-reported their relationship to the patient and the number of clinic visits they attended with the patient in the last 6 months. Clinicians self-reported their clinical role and specialty, number of patients seen with advanced ovarian cancer per month, and number of years spent caring for patients with advanced ovarian cancer.

#### Cognitive Interviews

We conducted individual, semistructured cognitive interviews with patients, caregivers, and clinicians in person and over Health Insurance Portability and Accountability Act–compliant video conferencing software (Multimedia Appendix 1). To identify potential usability challenges, participants were instructed to think aloud as they used the CASI in a testing environment that was not linked to the EHR. Trained interviewers (RAP, JB, and PB) observed participants, made note of any sections of the CASI that were difficult for participants to navigate, and invited participants to comment on aspects of the CASI’s content and design. Interviews with Spanish-speaking participants were conducted in Spanish by a bilingual member of this study’s team (JB). All participants reviewed patient- and caregiver-facing content, but only clinicians reviewed clinician-facing content. To identify potential barriers to implementing the CASI, interviewers asked participants to consider integration of the CASI into their existing routine, identify barriers to using the CASI regularly, and identify strategies to minimize patient, caregiver, and clinician burden. Interviews were audio recorded, professionally transcribed, and professionally translated from Spanish to English when applicable. Following cognitive interviews, participants completed a REDCap (Research Electronic Data Capture; Vanderbilt University) [27,28] survey that included quantitative measures of usability, acceptability, and burden. We refined the CASI in response to cognitive interview feedback.

#### Usability

The System Usability Scale (SUS) is a 10-item scale that assesses the usability of electronic systems. The SUS yields a single number representing a composite measure of the overall usability of the system being studied. Scores for individual items are not meaningful on their own. Total scores range from 0 to 100; higher scores indicate better usability [29], and scores of 70 or greater are acceptable [30].

#### Acceptability

The Acceptability of Intervention Measure (AIM) and Intervention Appropriateness Measure (IAM) are 4-item scales that each assess a single dimension of intervention acceptability. Response options range from 1 (“completely disagree”) to 5 (“completely agree”). Total scores for each measure range from 1 to 5. While cut scores have not yet been established for these measures, higher scores represent better acceptability [31].

#### Participant Burden

We assessed participant burden by asking participants to rate the extent to which they agreed with the statement “using the CASI placed a considerable burden on me.” Response options ranged from 1 (“strongly disagree”) to 5 (“strongly agree”).

### Analysis

#### Cognitive Interviews

We analyzed transcripts of cognitive interviews using directed content analysis [32]. Directed content analysis involves drawing key constructs from existing theory to develop an initial codebook. When one or more text segments are not represented in the initial codebook, the investigator creates new codes. Our initial codebook was based on the 2009 version of the CFIR domains and constructs. Author RAP, a nurse scientist with expertise in qualitative research, read and coded all transcripts in their entirety. To enhance trustworthiness and foster reflexivity, coding was reviewed by members of this study’s team with expertise in clinical research (JB) and health informatics (PB). Differences in data interpretation were resolved through discussion. We used MAXQDA 2022 (VERBI Software GmbH) to support qualitative data management.

#### Quantitative Measures

We summarized participant characteristics and SUS, AIM, and IAM scores using descriptive statistics. We performed analyses of variance to identify differences in SUS, AIM, and IAM scores across patients, caregivers, and clinicians and considered values of *P*<.05 significant.

## Results

### Participant Characteristics

Data collection took place between August 2023 and July 2024. Interviews lasted an average of 40 (SD 10) minutes. A total of 15 participants (5 patients, 5 caregivers, and 5 clinicians) completed this study. Characteristics of each participant group are detailed in Table 1. Briefly, participants were predominantly women (11/15, 73%), White (12/15, 80%), non-Hispanic (11/15, 73%), and working full- or part-time (9/15, 60%). Patients and caregivers were most commonly married or partnered (5/10, 50%) college graduates (5/10, 50%) and reported a range of annual household incomes: less than US $24,000 (2/10, 20%), US $75,000 to US $119,000 (2/10, 20%), or US $120,000 or more (2/10, 20%) per year. Caregivers identified as spouses or partners (2/5, 40%), parents (2/5, 40%), and friends (1/5, 20%) of patients. Caregivers had most often attended between 5 and 9 clinic visits with patients in the last 6 months (2/5, 40%). Clinicians were primarily physicians working in medical oncology (3/5, 60%) who reported caring for more than 20 patients with advanced ovarian cancer each month (5/5, 100%) and had 15‐19 years of experience caring for patients with advanced ovarian cancer (3/5, 60%).

**Table 1.** Participant characteristics.

			Total	Patients	Caregivers	Clinicians
All participants (N=15)						
	Age (years), mean (SD)		61 (13)	66 (5)	66 (19)	54 (16)
	Gender, n (%)					
		Woman	11 (73)	5 (100)	2 (40)	4 (80)
		Man	4 (27)	0 (0)	3 (60)	1 (20)
	Ethnicity, n (%)					
		Hispanic	4 (27)	2 (40)	2 (40)	0 (0)
		Non-Hispanic	11 (73)	3 (60)	3 (60)	5 (100)
	Race, n (%)					
		Asian	2 (13)	1 (20)	0 (0)	1 (20)
		Native American	1 (7)	1 (20)	0 (0)	0 (0)
		White	12 (80)	3 (60)	5 (100)	4 (80)
	Employment status, n (%)					
		Working	9 (60)	1 (20)	3 (60)	5 (100)
		Retired	4 (27)	2 (40)	2 (40)	0 (0)
		Disabled	2 (13)	2 (40)	0 (0)	0 (0)
Patients and caregivers (n=10)						
	Marital status, n (%)					
		Married or partnered	5 (50)	3 (60)	2 (40)	—^a^
		Single or never married	3 (30)	1 (20)	2 (40)	—
		Divorced	2 (20)	1 (20)	1 (20)	—
	Annual household income (US $), n (%)					
		Less than $24,000	2 (20)	2 (40)	0 (0)	—
		$45,000-$74,999	1 (10)	1 (20)	0 (0)	—
		$75,000-$119,000	2 (20)	1 (20)	1 (20)	—
		$120,000 or more	2 (20)	1 (20)	1 (20)	—
		Missing	3 (30)	0 (0)	3 (60)	—
	Educational attainment, n (%)					
		Did not graduate high school	2 (20)	2 (40)	0 (0)	—
		Graduated high school	1 (10)	0 (0)	1 (20)	—
		Graduated college	5 (50)	2 (40)	3 (60)	—
		Postgraduate degree	2 (20)	1 (20)	1 (20)	—
Caregivers (n=5)						
	Relationship to patient, n (%)					
		Spouse or partner	—	—	2 (40)	—
		Parent	—	—	2 (40)	—
		Friend	—	—	1 (20)	—
	Clinic visits attended with patient in last 6 months, n (%)					
		5‐9	—	—	2 (40)	—
		10‐14	—	—	1 (20)	—
		15‐19	—	—	1 (20)	—
		20 or more	—	—	1 (20)	—
Clinicians (n=5)						
	Role, n (%)					
		Physician	—	—	—	3 (60)
		Advanced practice nurse	—	—	—	2 (40)
	Patients seen with advanced ovarian cancer per month, n (%)					
		20 or more	—	—	—	5 (100)
	Clinical specialty, n (%)					
		Medical oncology	—	—	—	5 (100)
	Years caring for patients with advanced ovarian cancer, n (%)					
		10‐14	—	—	—	1 (20)
		15‐19	—	—	—	3 (60)
		20 or more	—	—	—	1 (20)

a Not applicable.

### Usability, Acceptability, and Burden

SUS, AIM, and IAM scores by participant group are reported in Table 2. Briefly, the overall mean SUS score was 72 (SD 16), which is above the threshold of acceptable usability [30]. The overall mean AIM score was 3.9 out of 5 (SD 1), while the overall mean IAM score was 4.1 out of 5 (SD 0.8). There were no statistically significant (*P*<.05) differences in SUS, AIM, or IAM scores across patients, caregivers, and clinicians. Moreover, 1/15 (7%) participants reported that the CASI was burdensome; this participant was a patient who experienced difficulty adjusting the font size on the CASI questionnaires during usability testing.

**Table 2.** Usability and acceptability of the Collaborative Agenda-Setting Intervention.

	Overall (n=15), mean (SD)	Patients (n=5), mean (SD)	Caregivers (n=5), mean (SD)	Clinicians (n=5), mean (SD)	*F* test (*df*)	*P* value
SUS^a^	72 (16)	64 (15)	78 (12)	73 (20)	0.82 (2)	.47
AIM^b^	3.9 (1)	3.8 (0.8)	4 (1)	4 (1.2)	0.05 (2)	.95
IAM^c^	4.1 (0.8)	4 (0.8)	4.2 (0.8)	4.2 (0.8)	0.07 (2)	.93

a SUS: System Usability Scale. Possible scores range from 0-100. Higher scores indicate greater usability, and scores ≥70 suggest above-average usability.

b AIM: Acceptability of Intervention Measure. Possible scores range from 1‐5. Higher scores indicate greater acceptability and appropriateness, respectively.

c IAM: Intervention Appropriateness Measure. Possible scores range from 1‐5. Higher scores indicate greater acceptability and appropriateness, respectively.

### Cognitive Interview Findings

#### Suggested Revisions

Participants made numerous suggestions to improve the clarity and helpfulness of CASI questionnaire items. For example, several participants suggested reducing the number of response options on the Control Preferences Scale from 5 to 3. Participants also identified several items they felt were redundant. Caregiver participants suggested explicitly instructing caregivers to respond on behalf of the patient. Clinician participants were concerned about patients and caregivers being “locked in” to a specific preference or decision. For example, these participants suggested the CASI ask patients and caregivers what topics they would like to discuss rather than giving them the opportunity to identify topics they would like to avoid. Furthermore, 1 clinician observed that this modification would allow clinicians to raise topics they believed needed to be addressed even if the patient or caregiver was not previously interested in discussing it. Finally, some participants wanted the option to request a follow-up phone call or to opt out of navigation (ie, a follow-up phone call that would arrange for them to see social work or chaplaincy). A detailed list of suggested and incorporated revisions is provided in Table 3.

**Table 3.** CASI^a^ revisions derived from cognitive interviews.

Participant recommendations	Number recommending (n)				Actions taken
	PT^b^	CG^c^	CL^d^	All, n (%)	
Rephrase items for clarity	5	4	2	11 (73)	Rephrased items to improve clarity.
Eliminate redundant items	4	0	1	5 (33)	Revised items to eliminate redundancy.
Provide an option for personal interaction	3	2	0	5 (33)	Added item that reads: “Would you like someone to contact you about your options for additional support?”
Avoid locking patients and clinicians into a preference or decision	0	1	2	3 (20)	Rephrased Control Preferences Scales to refer to “most decisions.” Removed items allowing patients to indicate they “never” want to talk about a specific topic or undergo a specific procedure.
Consider limiting administration to patients who are not on their first line of treatment	1	0	2	3 (20)	Planned: Will confirm with clinicians whether potential participants are appropriate for pilot RCT.^e^
Revise items to reduce anxiety	0	1	2	3 (20)	Reframed items to allow patients to indicate that they would like to discuss a certain topic with their clinician.
Account for different caregiving roles	1	1	0	2 (13)	Added option for caregiver respondent to report their name and relationship to the patient.
Administer every 3‐4 weeks	0	1	1	2 (13)	Planned: Will enroll participants receiving treatment every 3 weeks into pilot RCT.
Clarify instructions for caregivers	0	2	0	2 (13)	Added the following instructions: “The following questions should be answered from the patient’s perspective. Please respond on behalf of the patient.”
Account for preferences for in-person versus digital communication	1	0	0	1 (6.7)	Added item that reads: “Would you like someone to contact you about your options for additional support?”
Allow for preference tracking over time	0	1	0	1 (6.7)	Improved readability of provider-facing Epic flow sheet.
Ask an open-ended item about what is meaningful to the patient	0	1	0	1 (6.7)	Added open-ended item that reads “What does a good day look like to you?”
Ask what caregiver is going through	0	1	0	1 (6.7)	Added open-ended item that reads “What, if anything, can we do to help you support the patient?”
Consider removing the option to request exact numbers and statistics	0	0	1	1 (6.7)	Rephrased to “with a lot of detail” and “without a lot of detail.”
Differentiate between ongoing and new or acute concerns	0	0	1	1 (6.7)	Planned: In emails to clinicians, study team will highlight changes in concerns.
Ensure mechanism for prompt follow-up	0	0	1	1 (6.7)	Planned: Study team will provide first-line follow-up and will track time spent responding to patient concerns.
Alert clinicians to unmet needs or changes in CASI responses	0	0	1	1 (6.7)	Planned: In emails to clinicians, study team will highlight changes in concerns.
Make follow-up navigation optional	1	0	0	1 (6.7)	Added item that reads: “Would you like someone to contact you about your options for additional support?”
Prompt patients to consider code status discussion	0	0	1	1 (6.7)	Added “the care I would like to receive if my health worsens” as a possible discussion topic.
Reduce number of response options for items about shared decision-making and communication preferences	0	0	1	1 (6.7)	Reduced the number of response options on the Control Preferences Scales from 5 to 3. Revised items related to communication preferences.

a CASI: Collaborative Agenda-Setting Intervention.

b PT: patient.

c CG: caregiver.

d CL: clinican.

e RCT: randomized controlled trial.

#### Barriers to and Facilitators of Implementation

##### Overview

Participants identified barriers to and facilitators of implementing the CASI across 4 CFIR domains: outer setting, inner setting, innovation characteristics, and individual characteristics. Exemplary quotes for the CFIR constructs addressed by participants are provided in Table 4. Definitions of each construct as they pertain to the CASI are incorporated into the Results section below.

**Table 4.** Potential barriers to and facilitators of implementing the Collaborative Agenda-Setting Intervention.

CFIR^a^ domain and construct		Participants reporting, n (%)	Role in implementation	Exemplary quotes
Outer setting				
	Needs and resources of those served	15 (100)	Facilitator	“I think the tool worked nicely. Provocative, but I think helpful. I think some people-- there is my good friend with cancer who does not want to know anything, absolutely nothing, but what has to happen today-- and I am the one who wants to know the future. So I think it is helpful.” [Female patient, aged 62 years]
Inner setting				
	Compatibility	5 (33)	Facilitator	“I do think any way you can streamline information to actually come to the clinician will help a lot in terms of its usability.” [Female advanced practice nurse, 10‐14 years of experience]
	Available resources	5 (33)	Barrier	“I mean there is unfortunately a huge problem now with social work in that there are no social workers. We have one remaining social worker in GI; we have two social workers in GYN, but they are really strapped, and I have found meaningful social work contact almost impossible.” [Female medical oncologist, 20+ years of experience]
	Relative priority	3 (20)	Both	“There are very few patients who are like, ‘I feel great, and I don’t have any of these things,’ you know, but the things that they have are often the same and constant from visit to visit.” [Female medical oncologist, 15‐19 years of experience]
	Tension for change	3 (20)	Facilitator	“That would be helpful because you know that question about anxiety and stuff I mean I have anxiety, I have anxiety about the finances I am primarily the one that has been managing the finances and seeing us through all of this.” [Male spousal caregiver, age not reported]
	Networks and communications	1 (6.7)	Barrier	“I think it is important for us to know that the patients are approached so we expect what will happen because there are going to be questions which is fine.” [Male medical oncologist, 15‐19 years of experience]
Innovation characteristics				
	Complexity	12 (80)	Barrier	“It’s not clear to me whether it’s me you’re asking about or you’re asking about her, and I should be answering questions about her.” [Female friend, aged 79 years]
	Design quality and packaging	12 (80)	Barrier	“Okay… So now I should press here? Where should I press now?” [Female patient, aged 63 years]
	Relative advantage	5 (33)	Facilitator	“It is nice to know if what kind of person they are so I can start understanding their values.” [Female advanced practice nurse, 10‐14 years of experience]
	Adaptability	4 (27)	Both	“[One concern would be if] you can’t go do this other thing that you were supposed to because you made a [different] decision back then.” [Male adult child caregiver, age not reported]
	Trialability	4 (27)	Both	“And for a caregiver, how would you present this to them? I mean I’m certainly not on her patient [portal].” [Female friend, aged 79 years]
Individual characteristics				
	Other personal attributes	11 (73)	Barrier	“I like just to talk. I’m not a person that likes filling out answers, to be honest with you.” [Female patient, aged 61 years]
	Knowledge and beliefs about the innovation	3 (20)	Barrier	“Even if they are finished with treatment, the last thing they want to think about is a conversation about death and dying when they think they are cured.” [Female advanced practice nurse, 15‐19 years of experience]
	Self-efficacy	3 (20)	Both	“Would I know some of these things? Yes, perhaps, but I would not feel comfortable with [reporting them on the patient’s behalf].” [Female friend, aged 79 years]
	Individual stage of change	2 (13)	Barrier	“The only one that would make me change my management style would be the last one, which is, ‘I prefer to leave all treatment decisions to my doctor.’” [Female medical oncologist, 15‐19 years of experience]
	Individual identification with organization	1 (6.7)	Barrier	“I have other sources [of health care].” [Female friend, aged 79 years]

a CFIR: Consolidated Framework for Implementation Research.

##### Outer Setting

All participants (15/15, 100%) addressed the needs and resources of those served, referring to the extent to which the needs of end users are accurately known and prioritized by the CASI [22]. Comments related to this construct were overwhelmingly positive. Participants reported that the topics addressed by the CASI are important, and they appreciated that the CASI was brief and easy to use. Further, 1 caregiver was especially enthusiastic about being able to update his or the patient’s preferences as they evolve over time. However, 1 caregiver observed that not every caregiver has proxy access to the patient portal, and 1 clinician worried that asking about preferences for prognostic communication may exacerbate patients’ anxiety.

##### Inner Setting

In total 5 (33%) participants addressed the CASI’s compatibility, which refers to how well the CASI will fit into existing practice norms, workflows, and systems [22]. Overall, participants approved of the CASI’s integration into the clinical workflow. Clinicians especially appreciated having the opportunity to follow up on needs that may not be addressed during a visit and the ease with which they could populate their visit notes with patients’ and caregivers’ CASI questionnaire responses. Further, 5 (33%) participants discussed the available resources for implementing the CASI [22]. Each of these 4 clinicians and 1 caregiver were concerned there would not be enough staff or supportive care services available to address patient- and caregiver-reported concerns. Furthermore, 3 (20%) participants addressed the CASI’s relative priority, which refers to the importance of implementing the CASI relative to other innovations [22]. Moreover, 1 clinician was concerned about the amount of information clinicians already review before a visit. A second clinician noted that, despite the importance of addressing supportive care needs, patients’ medical needs would likely take priority. Additionally, 3 (20%) participants addressed tension for change, which refers to the degree to which stakeholders believe current practices need to change [22]. While none of these participants shared a negative opinion of current practice, each acknowledged there is room for improvement in certain aspects of care. For example, 1 patient described an experience of suboptimal communication that was distressing to her, while 1 clinician expressed that it would be helpful to know a patient’s decision control preferences. Finally, 1 (6.7%) participant addressed networks and communications, which refers to the nature and quality of formal and informal communication within an organization [22]. This clinician wanted to be sure they would be notified when a patient completed the CASI.

##### Innovation Characteristics

A total of 12 (80%) participants addressed the CASI’s design quality and packaging, which refers to the level of perceived excellence in how the CASI is bundled and presented [22]. Participants appreciated the clean, simple layout of the CASI. However, participants made several suggestions related to font size and screen layout. Further, 12 (80%) participants also addressed the CASI’s complexity. As noted above, participants identified items they felt were redundant or overly complex and made suggestions to clarify user instructions. Furthermore, 5 participants addressed the CASI’s relative advantage, which refers to the advantage of implementing the CASI versus an alternative solution [22]. Patients appreciated being able to report their concerns in addition to their preferences, and clinicians observed that the CASI addresses topics that are not captured in our institution’s existing patient-reported symptom questionnaire. Additionally, 4 (27%) participants addressed the adaptability of the CASI, which refers to the extent to which the CASI can be adapted, tailored, or refined to meet user’s needs [22]. Participants provided suggestions to enhance the CASI’s adaptability. For example, 2 patients suggested making follow-up navigation optional. Moreover, 4 (27%) participants addressed the CASI’s trialability and approved of the iterative process through which it is being developed and tested.

##### Individual Characteristics

A total of 11 (73%) participants identified personal attributes of end users that may influence adoption of the CASI. Specifically, participants emphasized the need to consider patients’ and caregivers’ overall literacy, digital literacy, preferences for face-to-face or digital communication, patients’ time since diagnosis, and caregivers’ caregiving role. Further, 3 (20%) participants expressed knowledge and beliefs about the CASI content that may influence its adoption. For example, as noted above, 1 clinician wondered whether items about prognostic communication would exacerbate patients’ anxiety. Furthermore, 3 (20%) participants addressed potential challenges related to patients’ and caregivers’ self-efficacy to complete the CASI. In addition, 1 clinician suspected that patients who need supportive care services often lack the self-efficacy to seek assistance because they are experiencing symptoms of depression. Besides 1 caregiver did not feel comfortable responding to questions on the patient’s behalf. Additionally, 2 (13%) participants addressed their individual stage of change as it pertains to adopting the CASI. These clinicians expressed that they were unsure of the extent to which they would change their practice style in response to patients’ or caregivers’ CASI responses. Finally, 1 (6.7%) participant addressed her identification with the organization that created the CASI. This caregiver expressed that while she appreciated being asked about her supportive care needs, she would turn to her primary care provider rather than the cancer center for assistance.

## Discussion

### Principal Results

The findings of our study suggest that the CASI is a usable and acceptable intervention to foster person-centered ovarian cancer care. Participants suggested several revisions that have since been incorporated into the CASI. Participants identified several facilitators of implementing the CASI, including that the CASI meets the needs of patients, caregivers, and clinicians; is well-designed; and is compatible with the existing workflow. Additionally, participants indicated that the CASI offers a relative advantage over usual care and that there is interest among stakeholders in standardizing the way supportive care needs are identified and documented.

Participants also identified potential barriers to implementing the CASI, and the CFIR provided a relevant organizing framework for these barriers. The most frequently identified barriers were the complexity of the CASI and the resources available to address patients’ and caregivers’ supportive care needs. Our approach to intervention development has preemptively addressed some of these potential barriers. According to the Expert Recommendations for Implementing Change taxonomy [33], intervention complexity can be managed by eliciting local knowledge, conducting small tests of cyclical change, and providing ongoing training. Accordingly, we have prioritized stakeholder engagement throughout the process of intervention development and have taken an iterative approach to refining and testing the CASI [17]. Our next planned study will assess the feasibility of conducting a full-scale efficacy trial of the CASI in a pilot randomized controlled trial (RCT). During this trial, we will aim to manage the perceived complexity of the intervention by training participants to use the CASI and providing written training materials and resources in advance.

To address the potential barrier of limited available resources, the Expert Recommendations for Implementing Change taxonomy recommends securing additional funding and developing resource sharing agreements [33]. In our planned pilot RCT, members of this study’s team will be responsible for following up with patients and caregivers who report unmet supportive care needs. We will track the frequency with which these needs arise and will document the resources (eg, time and personnel) needed to address them. Upon completion of the pilot RCT, we hope to have a more robust understanding of the resources that will be required to implement the CASI. These findings will inform our funding application for a subsequent efficacy trial and may eventually inform resource allocations to support the integration of the CASI into routine care.

### Strengths and Limitations

There is a growing recognition of the potential for EHR-integrated supportive care interventions to improve health outcomes among patients with advanced cancer. Leaders in communication research have specifically called for the development of interventions that integrate into the clinic workflow and are disseminated through existing mechanisms [34,35]. The CASI meets this need and was developed according to stakeholders’ needs and feedback [17]. In the current study, we conducted usability testing in a linguistically and socioeconomically diverse sample of patients with advanced ovarian cancer, caregivers, and clinicians. In addition, our study design was informed by an established implementation science framework [22]. Nevertheless, this was a single-site study with 15 participants, and the generalizability of our findings to other settings may be limited. Additional research is needed to assess the feasibility of conducting a full-scale efficacy trial of the CASI, to compare the effect of the CASI on outcomes to that of usual care, and to assess implementation of the CASI in diverse real-world settings.

### Comparison With Prior Work

To our knowledge, the CASI is the first EHR-integrated supportive cancer care intervention to use an agenda-setting approach. Our finding that the CASI is usable by and acceptable to patients, caregivers, and clinicians is consistent with prior studies of EHR-integrated supportive care interventions. In a sample of patients with heterogeneous cancer types and their caregivers, a biopsychosocial care need screening system had above-average usability [36]. Similarly, in a sample of patients with heterogeneous cancer types, a patient-reported symptom and needs monitoring system was usable and relevant [37]. In a sample of more than 100 health professionals, a centralized location for storing patients’ values, goals, and preferences was viewed positively [38]. Our study adds to these findings by demonstrating that patients’ and caregivers’ values, preferences, and supportive care needs can be assessed and communicated as part of the same intervention without sacrificing usability or acceptability.

Widespread implementation of EHR-integrated supportive care interventions has not yet been realized, and research describing barriers to and facilitators of implementing these interventions is limited. However, in an evaluation of patient perspectives related to implementing a patient-reported symptom and needs monitoring system, Lyleroehr et al [37] found that low clinician engagement and suboptimal communication about the intervention were key barriers to implementation. Similarly, Wickline et al [39] found that more than half of patients with advanced ovarian cancer using a remote symptom and quality of life monitoring system felt it was not obvious their clinician used the system’s reports. In both studies, patients valued having the option to speak directly to their care team about their concerns [37,39]. In our planned pilot RCT, we will attempt to mitigate these potential barriers by offering patients and caregivers follow-up phone calls, providing clinicians with a summary of patients’ and caregivers’ responses ahead of a scheduled visit, and offering to initiate referrals to reduce clinician burden.

### Conclusions

Agenda-setting is a novel approach to promoting person-centered care for individuals with ovarian cancer. Our findings suggest the CASI is usable and acceptable to patients, caregivers, and clinicians. Guided by an implementation science framework, we identified several barriers to and facilitators of implementing the CASI. In subsequent research, we will assess the feasibility of conducting an efficacy trial comparing the CASI to usual care.

## Supplementary material

10.2196/66801 Multimedia Appendix 1 Cognitive interview guides.

## Abbreviations

AIM: Acceptability of Intervention Measure
CASI: Collaborative Agenda-Setting Intervention
CFIR: Consolidated Framework for Implementation Research
EHR: electronic health record
IAM: Intervention Appropriateness Measure
RCT: randomized controlled trial
REDCap: Research Electronic Data Capture
SUS: System Usability Scale
