# Supplementary material for: Usability, Acceptability, and Barriers to Implementation of a Collaborative Agenda-Setting Intervention (CASI) to Promote Person-Centered Ovarian Cancer Care: Development Study
Source: JMIR Cancer. 2025 Mar 10;11:e66801. doi: 10.2196/66801 (PMC11913317; doi:10.2196/66801)
Supplement: Multimedia Appendix 1 [file cancer-v11-e66801-s001.docx]

*A Spanish-language version of the patient and caregiver interview guide is available upon request to rachel_pozzar@dfci.harvard.edu.*

## Table of Contents

[Table of Contents 2](#_Toc187134109)

[Patient and Caregiver Interview Guide 3](#_Toc187134110)

[Introduction 3](#_Toc187134111)

[Design-Related Questions 3](#_Toc187134112)

[Usability-Related Questions 3](#_Toc187134113)

[Summary Questions 3](#_Toc187134114)

[Closing 4](#_Toc187134115)

[Clinician Interview Guide 5](#_Toc187134116)

[Introduction 5](#_Toc187134117)

[Design-Related Questions 5](#_Toc187134118)

[Usability-Related Questions 5](#_Toc187134119)

[Summary Questions 5](#_Toc187134120)

[Closing 6](#_Toc187134121)

## Patient and Caregiver Interview Guide

### Introduction

The interviewer reviews study procedures, offers to answer questions, and obtains written informed consent. The interviewer informs the participant prior to pressing “record” on the audio recorder.

### Design-Related Questions

To be asked in relation to the patient- and caregiver-facing CASI, patient- and caregiver-facing question prompt list, and clinician-facing Patient-Centered Care Profile and CASI smart phrase output.

- What parts of the screen stand out to you?
- What part do you want to interact with first? Second?
- Does this screen look the way you were expecting?
- What, if anything, on this screen could you do without?
- What, if anything, is missing from this screen?
- What other thoughts or comments do you have about this screen?

### Usability-Related Questions

Imagine you are a patient preparing for a visit with your clinician and that you are using the CASI for the first time.

- Could you show me how you would log in to the CASI?
- How would you make note of your values and preferences?
- How would you indicate that you are concerned about nausea? …about insurance?
- How would you edit your list of priority concerns?
- How would you submit your responses to your clinician?
- Where would you look for the question prompt list?
- Where would you look for the teaching sheet(s)?

Imagine you are a patient preparing for a subsequent visit with your clinician.

- How would you update your Patient-Centered Care Profile?

### Summary Questions

- What are your thoughts about the CASI overall?
- How might the CASI fit into your existing routine, if at all?
- How does the CASI compare to other tools you have used?
- How well do you think the CASI will meet your needs, if at all?
- What challenges do you think a patient might face when using the CASI?
- Is there a need for the CASI?
- How confident are you that you will be able to use the CASI?
- What other suggestions or comments do you have about the CASI?

### Closing

The interviewer thanks the participant for his or her time and stops audio recording.

## Clinician Interview Guide

### Introduction

The interviewer reviews study procedures, offers to answer questions, and obtains written informed consent. The interviewer informs the participant prior to pressing “record” on the audio recorder.

### Design-Related Questions

To be asked in relation to the patient- and caregiver-facing CASI, patient- and caregiver-facing question prompt list, and clinician-facing Patient-Centered Care Profile and CASI smart phrase output.

- What parts of the screen stand out to you?
- What part do you want to interact with first? Second?
- Does this screen look the way you were expecting?
- What, if anything, on this screen could you do without?
- What, if anything, is missing from this screen?
- What other thoughts or comments do you have about this screen?

### Usability-Related Questions

Imagine you are preparing for a visit with your patient and that you are using the Patient-Centered Care Profile and CASI smart phrase for the first time.

- How would you review the patient’s Patient-Centered Care profile?
- How would you populate the visit note with the patient’s CASI responses?
- How would you assess how the patient is feeling?
- How would you identify the patient’s priority concerns?
- Where would you find the tailored recommendations and resources?

### Summary Questions

- What are your thoughts about the CASI overall?
- How does the CASI compare to other similar existing tools in your setting?
- What kind of changes do you think will need to be made for the CASI to work effectively in your setting?
- How well do you think the CASI will meet the needs of your patients, if at all?
- How, if at all, does the culture here impact the implementation of the CASI?
- Is there a strong need for the CASI?
- How well does the CASI fit with the values, norms, and routines of your practice?
- To what extent might the CASI take a back seat to other projects right now?
- Do you expect to have sufficient resources to use the CASI?
- How confident are you that you will be able to use the CASI?
- What other suggestions or comments do you have about the CASI?

### Closing

The interviewer thanks the participant for his or her time and stops audio recording
